# Opinion *versus* practice regarding the use of rehabilitation services in home care: an investigation using machine learning algorithms

**DOI:** 10.1186/s12911-015-0203-1

**Published:** 2015-10-09

**Authors:** Lu Cheng, Mu Zhu, Jeffrey W. Poss, John P. Hirdes, Christine Glenny, Paul Stolee

**Affiliations:** 1 Department of Statistics and Actuarial Science, University of Waterloo, 200 University Avenue West, Mathematics Building No. 3, Waterloo, ON N2L 3G1 Canada; 2 School of Public Health and Health Systems, University of Waterloo, 200 University Avenue West, Waterloo, ON N2L 3G1 Canada

**Keywords:** Machine learning, interRAI, Rehabilitation, Home care

## Abstract

**Background:**

Resources for home care rehabilitation are limited, and many home care clients who could benefit do not receive rehabilitation therapy. The interRAI Contact Assessment (CA) is a new screening instrument comprised of a subset of interRAI Home Care (HC) items, designed to be used as a preliminary assessment to identify which potential home care clients should be referred for a full assessment, or for services such as rehabilitation. We investigated which client characteristics are most relevant in predicting rehabilitation use in the full interRAI HC assessment.

**Methods:**

We applied two algorithms from machine learning and data mining ― the LASSO and the random forest ― to frequency matched interRAI HC and service utilization data for home care clients in Ontario, Canada.

**Results:**

Analyses confirmed the importance of functional decline and mobility variables in targeting rehabilitation services, but suggested that other items in use as potential predictors may be less relevant. Six of the most highly ranked items related to ambulation. Diagnosis of cancer was highly associated with decreased rehabilitation use; however, cognitive status was not.

**Conclusions:**

Inconsistencies between variables considered important for classifying clients who need rehabilitation and those identified in this study based on use may indicate a discrepancy in the client characteristics considered relevant in theory versus actual practice.

## Background

Rehabilitation interventions that target older adults have the potential to generate widespread health benefits. The goal of these interventions is to help individuals restore their functional ability or to maintain their residual functional capacity. A contemporary meta-analysis of randomized controlled trials demonstrated significant short and long term improvements associated with inpatient geriatric rehabilitation related to functional status, admission to nursing homes and mortality [1]. In addition, rehabilitation in home-based settings can provide effective therapy for this population leading to system improvements including decreased costs, more appropriate resource use, and avoidance of institutional placements [2–7]. Despite these well-established benefits, multiple challenges in providing service to this group exist. For older adults, increased levels of frailty, a higher burden of comorbid disease and multi-causal disease etiology all contribute to increasing the complexity of care for older rehabilitation clients [8–11].

The elevated risk of hospitalization due to functional decline within this population [8, 12] has resulted in an effort to investigate the feasibility and effectiveness of rehabilitation for older persons in home-based settings [2–7, 13]. A primary goal of providing rehabilitation services within the home (through physical therapy or occupational therapy or a combination of both) is to allow individuals to maintain or improve physical functioning, quality of life and their overall independence while remaining in the community longer [14]. Stolee and colleagues [13] conducted a systematic literature review to compare inpatient versus home-based rehabilitation for older adults with musculoskeletal disorders. The studies reviewed consistently suggested that home-based rehabilitation was either equal to or better than hospital based rehabilitation despite the wide variety of outcomes considered.

Home care has been described as the “next essential service” [15] and is an increasingly important component of the health care systems in Canada and elsewhere. Driving this growth is the view that a comprehensive approach to home and community care could lead to a more sustainable health care system, and will drive benefits in other key priority areas including a reduction in hospital wait times [16]. However, currently only 1 in 10 Canadians aged 65 years or older receive formal home care services each year in Canada, and of those, 19 % report unmet needs [17]. In addition, close to three-quarters of home care clients who have been identified as having rehabilitation potential do not receive any type of rehabilitation therapy [18]. With an increased emphasis on policies surrounding “aging in place” initiatives [19], and evidence of real and self-perceived unmet needs in home care [20], it is necessary to consider how to allocate limited resources to clients who are most likely to benefit from home-based services such as rehabilitation [21].

In order to bridge the gaps between service need, provision and use, it is essential to identify the key factors that predict successful rehabilitation as well as understand how this limited resource is currently being allocated. Due to the challenging nature of older rehabilitation clients and considerable variability even within specific diagnostic categories [10, 11, 22], gathering this information requires high quality and comprehensive client data. Standardized assessment systems, such as the interRAI assessment tools [23–25], are leading source of these data. The interRAI consortium is an international organization of researchers leading the development of a suite of standardized assessment systems for use in many health care settings [26]. The interRAI Home Care (interRAI HC) [27] assessment system is mandated for use with all longer stay adult, non-palliative home care clients in Ontario [9], and is used to inform and guide comprehensive care and service planning in community-based settings.

Recently, the interRAI Contact Assessment (interRAI CA, or simply CA) [28] was created using a subset of interRAI HC items as a preliminary assessment of home care clients. The aim of the CA is to identify persons requiring comprehensive assessment and to identify the urgency of need for a number of services, including rehabilitation. The CA uses a variety of activities of daily living (ADLs, e.g., dressing), instrumental activities of daily living (IADLs, e.g., housework) and cognition items in its Rehabilitation Algorithm (RA), which is intended to flag suitability for rehabilitation services. The items selected for inclusion in the RA were chosen by consultation with clinicians and other experts with the intention to aid home care intake staff to identify clients that may benefit from rehabilitation. As such, items included in the RA algorithm are strongly indicative of what experts believe are the most important client characteristics for determining rehabilitation needs, as well as how limited rehabilitation resources should be allocated.

An interesting question that arises, therefore, is whether these expert beliefs match actual clinical practices. The aims of our study were to use tools from machine learning and data mining to identify items from the interRAI HC instrument that are most predictive of whether a client will receive rehabilitation services in actual clinical practice, and to compare them with items selected by experts for the RA. By contrasting the items on these two lists, we are able to describe and highlight key differences between expert opinion and actual clinical practice.

## Methods

The present study is a component of a larger multidisciplinary health research program – “InfoRehab” - that aims to improve the understanding and use of health information for musculoskeletal rehabilitation clients (see, e.g., [29, 30]). A primary aim of InfoRehab is to address questions surrounding the role of rehabilitation in home care through advanced statistical analysis of large databases of health information (including RAI-HC data).

Ethics clearance was granted for this study from the University of Waterloo Office of Research Ethics (ORE Reference #14795). This research involved a secondary analysis of data collected for clinical and administrative purposes. The RAI-HC and CA data are part of current practice, and are collected as part of the intake process or as part of a routine re-assessment of ongoing needs. The identities of the individuals in the provincial RAI-HC dataset were unknown to the researchers. No individual consent was obtained, as it would be impractical for the province’s publicly-funded home care organizations to attempt to obtain written consent from over 130,000 clients. No personally identifying information was available in the dataset used for analysis. Social Insurance Number or provincial Health Card Number were not available in the dataset, nor any other identifiers that would enable the researchers to identify an individual. The identifiers included in the dataset are provided by the province’s Community Care Access Centres (which coordinate publicly funded home care services in the province) and are of one of two types: either an internal case record number used only for internal tracking, and not a ‘real world’ identifier, or an encrypted health card number. These numbers are consistent so we can track longitudinal cases, but they are not useful in personally identifying individuals or linking to other information that would do so. Results of the research are reported only at an aggregate level, and no individual-level information is reported.

### Instrument

The international research consortium interRAI is a 32-country, collaborative, not-for-profit network of researchers and clinicians focused on the development and application of innovative health assessment systems that support evidence-informed decision making at all levels of health and social service delivery. The interRAI consortium has developed 12 comprehensive assessment instruments specifically designed for use with complex populations across the health care continuum [24, 25, 31]. In general, interRAI instrument development occurs through a number of steps that include reviewing current literature, consulting with experts and applying statistical analyses. The RAI-HC assessment instrument is used to collect detailed health and functional information on home care clients in Ontario, Canada and other jurisdictions [27]. It contains over 300 items measuring cognition, mood, psychosocial issues, nutrition and physical functioning and other client characteristics. Examples of its current uses include care planning, outcome measurement and quality indicators [24, 25, 31]. Since 2002 in Ontario, full interRAI-HC assessments are mandated for use with all longer stay home care clients (i.e., those expected to receive services for at least 60 days; approximately 50 % of the overall provincial publicly-funded home care case load). Follow-up assessments are completed every 6 months or earlier in the event of major clinical changes.

The CA is a standard preliminary assessment developed for use as a screening tool during adult home care intake that guides initial service planning and decision making. It was specifically designed to be completed during initial contact with potential home care clients and includes a subset of items (approximately 50) from the RAI-HC. The purpose of the CA is to identify patients who require a full assessment using the RAI-HC, those with urgent needs for nursing or personal support services, and those for whom a referral for rehabilitation may be appropriate [9]. The development of the CA was guided by the interRAI Instrument and Systems Development (ISD) group in conjunction with the development of the new standardized suite of interRAI instruments.

**Fig. 1:**
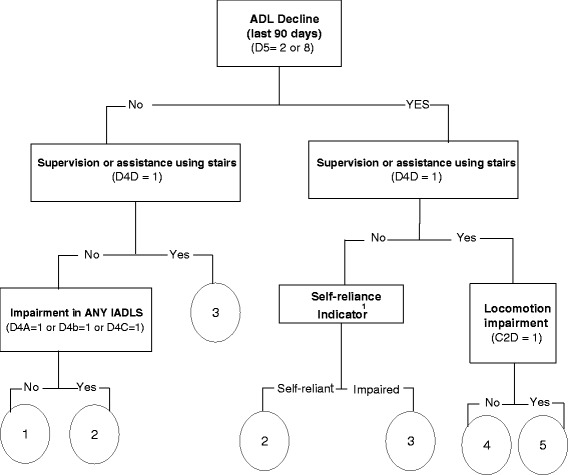
RAI CA rehabilitation algorithm. This figure and related notes are adapted from the interRAI Contact Assessment (CA) Form and User’s Manual [27] The Rehabilitation Algorithm yields a score from 1 to 5, with higher scores indicating that the clients is more likely to need rehabilitation services. ^1^Definition in text

To inform referrals for rehabilitation, the CA contains a decision tool called the RA. Development of the algorithm was based on case manager ratings of who they felt would be a candidate for rehabilitation services, as well as actual receipt of physiotherapy or occupational therapy following screening. Decision tree models were used to construct a decision support algorithm that combines the findings of various domains into a single summary measure that can be used to inform decision making. It is based on a small number of variables relating to a recent decline in ability to perform activities of daily living, functional and mobility status, and cognitive impairment. Figure 1 shows the decision tree representation for the RA. The algorithm is made up of both individual items and one summary scale – the Self Reliance Index [28]. Overall, the algorithm categorizes potential clients into five groups based on how likely they will need rehabilitation services. The purpose of the algorithm is to aid home care workers in identifying clients that may benefit from rehabilitation services, recognizing that these decisions will also be informed by clinical judgement, resource constraints, and individual circumstances.

This figure and related notes are adapted from the interRAI Contact Assessment (CA) Form and User’s Manual [28]. The Rehabilitation Algorithm yields a score from 1 to 5, with higher scores indicating that the clients is more likely to need rehabilitation services. A person is classified as “impaired” on the self-reliance indicator if any of the following characteristics are present: modified independent or any impairment in cognitive skills for daily decision making (C1 = 1); received supervision or any physical help in one or more of the assessed ADLs [bathing (C2a = 1), personal hygiene (C2b = 1), dressing lower body (C2c = 1), and/or locomotion (C2d = 1)]. The Rehabilitation Algorithm (Fig. 1) was derived using a research dataset of ~ 500 screened adults on entry to Ontario home care in 2005, as part of a pilot of the interRAI Contact Assessment. It used interactive decision tree modeling, in SAS, with clinical experts used to guide decision points and weigh alternate splits/constructions.

### Subjects

The data consisted of initial RAI-HC (version 2.0) assessments for 135,184 newly admitted home care clients evaluated in the community between October 2005 and March 2008. These data were linked to service records that contained information regarding home care service usage during the same period. Rehabilitation (outcome variable) was indicated by whether the client received physiotherapy or occupational therapy within 6 months of their initial assessment, which was coded as a binary indicator.

Of the available items in the RAI-HC, a total of 239 were utilized as potential predictors in the analysis. Items were dropped if they were regarded by the authors to be clearly unrelated to rehabilitation. Items that were clear prompts for rehabilitation (e.g., scheduled physiotherapy) were also dropped. The items used were primarily treated as categorical variables as indicated in the instrument; only number of falls (K5), number of visits to the hospital (P4A), emergency room and/or emergent care use (P4B and P4C), number of medications (Q1) and age were treated as continuous variables.

### Analysis

The statistical analysis was performed in two main steps.

#### Matched sampling

Considering that the CA was designed to be used during first contact with potential home care clients to determine those who would receive a full assessment (and who would not), and that the RAI-HC is used with those who are expected to or have received services for at least 60 days (long stay clients), the clients in the RAI-HC database may be different from the CA target population. Therefore, items deemed most predictive of service utilization based on long stay clients who received the RAI-HC assessment may not serve as an ideal benchmark for the broader CA target population of home care referrals. To address this issue, we created a frequency-matched dataset [29], by taking stratified samples from the RAI-HC database in such a way that the joint distribution of five key variables was the same in our sample as that in the CA database. The five key variables used to match the two groups were: age, gender, impairment in activities of daily living, cognition, and falls. The parameters used to define these five variables were identified in Table 1 as follows: Gender = 1 if male; ADL = 1 if any ADL item (H2A-H2J) was ≥ 1; Cognitive skills = 1 if cognitive skills for daily decision making (B2A) ≥ 1, Falls = 1 if falls frequency (K5) ≥ 1. For example, Table 1 shows that 0.06 % of the CA population were under 50 years of age, male (Gender = 1) with ADL = 1, Cognition = 1, and falls frequency ≥ 1 (Falls = 1). So, if we take a sample of 10,000 from the RAI-HC data, we will include exactly 10,000 × 0.06 % = 6 individuals with such characteristics.

**Table 1:** Percent of clients in the interRAI-CA population defined by the five matching variables

				Age (%)					Total
Gender	ADL	Cognition	Falls	<50	50–64	65–74	75–84	> 84	(%)
1	1	1	1	0.06	0.16	0.15	0.20	0.09	0.66
1	1	1	0	0.39	0.41	0.73	2.12	1.68	5.33
1	1	0	1	0.39	0.60	0.38	0.36	0.10	1.83
1	1	0	0	0.72	1.57	2.00	3.57	2.12	9.98
1	0	1	1	0.09	0.11	0.05	0.08	0.01	0.34
1	0	1	0	0.14	0.18	0.23	0.62	0.44	1.61
1	0	0	1	2.09	1.79	0.72	0.44	0.09	5.13
1	0	0	0	3.32	4.41	3.76	4.37	1.84	17.70
0	1	1	1	0.05	0.13	0.11	0.24	0.11	0.64
0	1	1	0	0.41	0.46	0.69	2.82	3.56	7.94
0	1	0	1	0.46	0.70	0.48	0.56	0.16	2.36
0	1	0	0	1.23	2.44	2.99	6.36	5.02	18.04
0	0	1	1	0.07	0.08	0.05	0.09	0.03	0.32
0	0	1	0	0.16	0.15	0.23	0.89	0.70	2.13
0	0	0	1	1.96	1.43	0.56	0.47	0.10	4.52
0	0	0	0	4.62	4.84	3.81	5.39	2.81	21.47
Total				16.16	19.46	16.94	28.58	18.86	100.00

#### Variable selection and ranking

There are several machine learning techniques that can be used for selecting key predictors from large datasets with many variables – recently, these techniques have been utilized in a variety of health-related applications [31–37]. The main variable-selection tool used in this study is called the LASSO [38]. It has been the most studied variable-selection tool over the last decade. Many variations now exist [39, 40].

To describe what the LASSO does, we use standard mathematical notation,

$$ \mathbf{y}=\left[\begin{array}{c}\hfill {y}_1\hfill \\ {}\hfill \vdots \hfill \\ {}\hfill {y}_n\hfill \end{array}\right],\kern1em \mathbf{X}=\left[\begin{array}{ccc}\hfill {x}_{11}\hfill & \hfill \cdots \hfill & \hfill {x}_{1d}\hfill \\ {}\hfill \vdots \hfill & \hfill \ddots \hfill & \hfill \vdots \hfill \\ {}\hfill {x}_{n1}\hfill & \hfill \cdots \hfill & \hfill {x}_{nd}\hfill \end{array}\right],\kern1em \boldsymbol{\upbeta} =\left[\begin{array}{c}\hfill {\beta}_1\hfill \\ {}\hfill \vdots \hfill \\ {}\hfill {\beta}_d\hfill \end{array}\right], $$

where *y*_*i*_ ∈ {0, 1} is a binary indicator for the outcome; *x*_*ij*_ is the *j* -th predictor variable for subject *i*; and *β*_*j*_ is the *j* -th regression coefficient. Let *l*(**β**; **X**, **y**) denote the log-likelihood function based on modeling each *y*_*i*_ as a Bernoulli random variable with parameter, *p*_*i*_≡ Pr (*y*_*i*_ = 1), and linking *p*_*i*_ to the predictors *x*_*i*1_, *x*_*i*2_, ⋯, *x*_*id*_ by the logistic equation,

1 $$ \log \frac{p_i}{1-{p}_i}={x}_{i1}{\beta}_1+{x}_{i2}{\beta}_2+\cdots +{x}_{id}{\beta}_d. $$

Unlike classical logistic regression that estimates the regression coefficients by maximizing *l*(**β**; **X**, **y**), the LASSO accomplishes this by solving the following optimization problem:

2 $$ \underset{\boldsymbol{\upbeta}}{ \max}\kern1em l\left(\boldsymbol{\upbeta}; \mathbf{X},\mathbf{y}\right)-\uplambda \Omega \left(\boldsymbol{\upbeta} \right), $$

where *l*(**β**; **X**, **y**) is the log likelihood function, and

$$ \Omega \left(\boldsymbol{\upbeta} \right)={\displaystyle \sum_{j=1}^d}\left|{\beta}_j\right| $$

is a penalty function that shrinks the regression coefficients *β*_1_, *β*_2_, ⋯, *β*_*d*_ and forces some of them to become zero. Consequently, only the predictors with regression coefficients that are nonzero will be “selected” by the model that the LASSO produces.

Fewer predictors will be selected (more coefficients will become zero) as the non-negative parameter, λ (which controls the amount of shrinkage), is increased. The choice of λ largely controls how many predictors are selected, and therefore must be carefully justified.

**Fig. 2:**
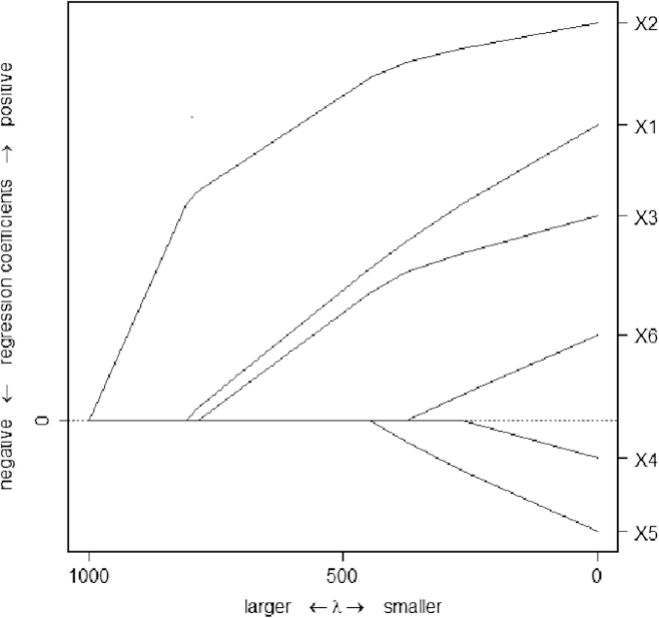
A toy example illustrating the solution path from LASSO. There are six predictors, ***X***_1_, …, ***X***_6_. When ***λ*** is very large, all six coefficients are forced to be zero. As ***λ*** decreases, the coefficients become nonzero (and the predictors enter the model) in the following order: ***X***_2_, ***X***_1_, ***X***_3_, ***X***_5_, ***X***_6_, ***X***_4_

As we have described elsewhere [41], to circumvent this “inconvenience”, we took into account not just one solution to the optimization problem (2) – given by a particular, possibly subjective, choice of λ – but the entire solution path [42] as λ changed. With a sufficiently large λ, all regression coefficients are forced to be zero and no predictor variable is selected. The regression coefficients become nonzero and predictor variables enter the model sequentially as λ is gradually decreased, as illustrated by a toy example in Fig. 2. The order in which predictor variables enter the model was used to *rank* their relative importance. In the toy example of Fig. 2, there are six predictors, X1,…, X6. When λ is very large, all six coefficients are forced to be zero. As λ decreases, the coefficients become nonzero (and the predictors enter the model) in the following order: X2, X1, X3, X5, X6, X4.

To obtain a more stable ranking, we followed an “ensemble approach” [43, 44], instead of ranking all the variables just once using the entire data set (*N* = 135,184). We first drew 100 frequency-matched samples (see earlier Section on “Matched Sampling”) from the data set, *S*_1_, *S*_2_, …, *S*_100_, each of size *n* = 10, 000. Let *r*(*j*, *k*) denote the rank of variable *j* based on sample *S*_*k*_. For each variable *j*, we then calculated its average rank over the 100 samples,

3 $$ \overline{r}(j)=\frac{1}{100}{\displaystyle \sum_{k=1}^{100}}r\left(j,k\right), $$

as well as *σ*(*j*), the standard deviation of $\overline{r}(j)$. We did not include interaction terms in our logistic model (1) – only main effects were considered. With 239 main effects, there would have been “239 choose 2” or 28,441 potential two-way interaction effects alone (not to mention any higher-order interactions), and it became practically infeasible for us to run the LASSO (and obtain the entire solution path) with this many variables.

As an indirect way to account for potential interaction effects, we recalculated $\overline{r}(j)$ and *σ*(*j*), this time relying on the variable importance measure (VIM) from Breiman’s random forest (RF) algorithm [45] to define each *r*(*j*, *k*). The RF algorithm essentially fits a collection of decision trees, which model interaction effects automatically [46]. The variable importance measure produced by the random forest, or simply RF-VIM, is based on *marginal* evaluations of the would-be deterioration in the model’s overall performance had the values of a predictor been permuted [47] ― the rationale being that, if permuting the value of a predictor does not have much effect on the model’s performance, it must not be a very important predictor, and vice versa. Hence, two variables may score high on the RF-VIM scale due to a certain interaction between them having a significant effect on the outcome, though the VIMs will not reveal that this is the reason for the high scores.

### Software

We used two R packages, “grplasso” and “randomForest”, to compute *r*(*j*, *k*) as described above. The algorithm implemented in “grplasso” is actually a variation of the original LASSO, called the “group LASSO” [48]. We used this variation because many of our predictors were categorical. A categorical predictor is often coded by a number of dummy variables in regression analysis, and the group LASSO forces these dummy variables to enter or exit the model together as a group along the solution path.

## Results

### Matched sampling

**Fig. 3:**
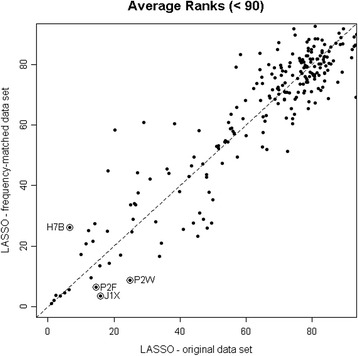
Average ranks from the LASSO. Results from the original data set versus those from the frequency-matched data set. There is good agreement for most of the top-ranked variables, other than the ones circled, which we discuss in the text

Table 1 shows the percent of clients in the CA population in each subcategory partitioned by the five matching variables. Figure 3 is a plot of the $\overline{r}(j)$’s obtained by running the LASSO on the original data set against those obtained from running it on the frequency-matched data set. There is good agreement for most of the top-ranked variables, other than the ones circled, which we discuss below.

Most points were close to the 45° line, which meant that, for the majority of variables, the two sets of average ranks were in good agreement whether the original data or the frequency-matched data were analyzed. This was especially true for the top ranked variables (bottom left corner). There were a small number of outlying points. In particular, the variables - chemotherapy (P2F), cancer present in past five years (J1X) and nurse monitoring less than daily (P2W) - appeared more important on the frequency-matched data set, whereas the variable - caregivers believe client is capable of increased functional independence (H7B) - appeared more important on the original data set. These differences may be attributable to the greater medical complexity expected in longer term home care clients assessed with the RAI-HC. From this point on, we focused on the frequency-matched data set only.

### Variable selection and ranking

**Fig. 4:**
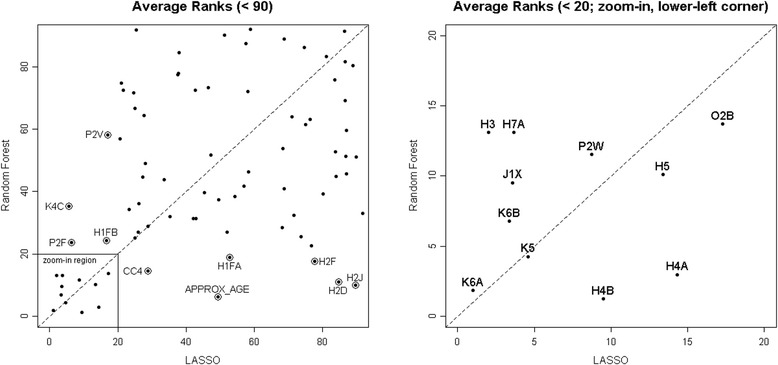
Average ranks from the LASSO versus those from the RF, both using the frequency-matched data set. The right panel is a zoom-in version of the bottom-left corner in the left panel, so that these variables, which both LASSO and RF assigned an average rank of higher than 20, can be labeled

Figure 4 plots the $\overline{r}(j)$’s obtained by the LASSO against those obtained by the RF. The right panel is a zoom-in version of the bottom-left corner in the left panel, so that these variables, which both LASSO and RF assigned an average rank of higher than 20, can be labeled.

Variables in the upper-right region of the plot received relatively low average ranks from both the LASSO and the RF, and therefore were not considered to be important predictors of rehabilitation use in this population. Variables in the upper-left region were more important for the LASSO than for the RF; these included pain intensity disrupts usual activity (K4C), chemotherapy during last 7 days (P2F), shopping difficulty (H1FB), and daily nurse monitoring (P2Y). Similarly, those in the lower-right region were more important for the RF than for the LASSO; these included time since last hospital stay (CC4), approximate age (AGE), shopping performance (H1FA), ADL-dressing lower body (H2F), ADL-locomotion outside of home (H2D), and ADL-bathing (H2J). These differences most likely came about because we did not consider any interaction effects in the LASSO approach, whereas in the RF approach interaction effects were automatically considered. The variables in the lower-left region are the most relevant ones to this investigation. They were identified to be strong predictors of rehabilitation service use by both the LASSO and the RF. Results about these variables are summarized in Table 2. The fourth column (effect on odds ratio) indicates whether the variables are predictive of increased rehabilitation use (+) or decreased rehabilitation use (-).

**Table 2:** Top-ranked variables identified by both the LASSO and the random forest

Variable		LASSO			Random forest	
		Average rank	(Standard deviation)	Effect on odds ratio	Average rank	(Standard deviation)
K6A	Unsteady gait	1.00	(0.00)	+	1.82	(0.05)
H3	ADL decline	2.02	(0.01)	+	13.11	(0.49)
K6B	Limits going outdoors due to fear of falling	3.38	(0.15)	+	6.76	(0.23)
J1X	Cancer (last 5 years)	3.58	(0.13)	-	9.52	(0.38)
H7A	Client believes can improve	3.68	(0.07)	+	13.11	(0.47)
K5	Falls frequency	4.59	(0.16)	+	4.26	(0.07)
P2W	Nurse monitoring < daily	8.75	(0.26)	-	11.53	(0.38)
H4B	Mode of locomotion – outdoors	9.49	(0.11)	+	1.24	(0.04)
H5	Stair climbing	13.39	(1.34)	+	10.09	(0.33)
H4A	Mode of locomotion – indoors	14.30	(0.45)	+	2.96	(0.03)
O2B	Better off in different environment	17.26	(0.29)	-	13.37	(0.52)

Table 3 lists the 11 top-ranked RAI-HC variables identified using the machine learning techniques (variables in the bottom-left corner of Fig. 4) and the 10 items used in the RA. Both lists contain ADL decline (RAI-HC, item H3; CA, item D5) and stair climbing (RAI-HC, item H4B; CA, item D4D). Items that appear in both columns are bolded; D4A, D4B, and D4C (marked by †) are part of the IADLs; whereas C1, C2A, C2B, and C2C (marked by ‡) are part of the self-reliance measure.

**Table 3:** Comparing top ranked RAI-HC items with items in the CA rehabilitation algorithm

Top ranked items by the LASSO and the RF		Items included in the CA rehabilitation algorithm	
K6A	uUnsteady gait	D5	ADL decline
H3	ADL decline	D4D	Stair climbing
K6B	Limits going outdoors due to fear of falling	D4A†	Meal preparation
JIX	Cancer (last 5 years)	D4B†	Managing housework
H7A	Client believes can improve	D4C†	Managing medications
K5	Falls frequency	C1‡	Skills of daily decision making
P2W	Nurse monitoring < daily	C2A‡	Bathing
H4B	Stair climbing	C2B‡	Personal hygiene
H5	Mode of locomotion - indoors	C2C‡	Dressing lower body
H4A	Mode of locomotion – outdoors	C2D	Locomotion
O2B	Better off in different environment		

Six of the ten items identified using the machine learning approach were related to the clients’ ability to ambulate – a client having increased falls frequency (K5), in danger of falling (K6B) or using an assistive device (such as a cane or walker) as their primary mode of locomotion (H5, H4A) were all positive indicators of rehabilitation service use. The four additional items selected by the machine learning approach included diagnosis of cancer over the past five years (J1X), client or primary caregiver feels that client would be better off in another living environment (O2B), and nurse monitoring less than once per day (P2W) as negative indicators; and clients belief that they can improve (H7A) as a positive indicator of rehabilitation service use. The list of top ranked variables using the machine learning techniques did not include any items specifically related to the client’s cognitive status.

## Discussion

This study builds on our previous explorations using machine learning algorithms to predict and to understand rehabilitation service use [33, 34], with the overall aim of developing methods to better target limited rehabilitation resources. Specifically, we compared expert opinions manifested in the rehabilitation algorithm associated with the CA with client characteristics that actually drove clinical practice in Ontario during the period of 2005–2008.

Since the CA and the RAI-HC are targeted at different populations of home care clients, we sampled a sub-population of the RAI-HC clients in such a way that matched their general characteristics to the population receiving the CA. To investigate the effect of this matching exercise, we repeated the LASSO part of our analysis using the original (unmatched) RAI-HC data. There were no major discrepancies among the top variables identified, other than a few items that may be attributable to the greater medical complexity expected in longer-term home care clients that are typically assessed by the RAI-HC. This matching approach could be used with other health information system databases to compare clients who are on a caseload with those in a screening database.

Overall, we found that actual clinical practices and expert opinions do overlap to a certain degree. For example, there is clear agreement that the abilities to climb stairs and to move about are two key factors that determine whether a patient is recommended for rehabilitation services. But we also found some differences. Some of the variables identified by the machine learning algorithms as predictive of rehabilitation use – such as falls frequency, unsteady gait, or limits in going outdoors – could be considered in future versions of the rehabilitation algorithm. This might lead to improved rehabilitation outcomes – for example, rehabilitation interventions that prevent falls could also reduce adverse outcomes such as injury, hospitalization or death.

We found that neither the LASSO nor the RF specifically identified cognitive impairment as a strong predictor associated with rehabilitation service utilization. The RA, on the other hand, applies cognitive impairment through the Self Reliance Index – in one of the later splits in the decision tree – which results in higher priority access to rehabilitation for those with cognitive impairment. Others have classified cognitive impairment as a significant negative predictor for rehabilitation potential and/or achievement in older patients [49]. A rationale for this is that impaired cognition will inhibit adherence to instructions for therapy and exercise programs. On the other hand, clinicians have also been able to show that patients with lower cognitive function could improve with access to rehabilitation [50–53]. Perhaps the LASSO and RF techniques did not find cognitive impairment to be predictive because other variables, such as client belief in potential for improvement, are acting as proxies for adequate cognitive function. Alternatively, it may be that there is limited use of cognitive impairment as a criterion in practice to allocate rehabilitation services. We note that in other investigations by our group using the RAI-HC data, we found that a clinical diagnosis of dementia was associated with less likelihood of receiving rehabilitation services [29]. This apparent discrepancy may be due to the differences in the samples and/or analytical approaches between the two studies (i.e., use of a “matched” versus a complete sample). It may also be due to the differences between the two variables used – an observed loss of cognitive capacity (the cognitive skills for daily decision-making variable used in the RA) may not influence a decision to provide or limit rehabilitation, while an explicit diagnosis of dementia might be more influential.

The variable, ‘diagnosis of cancer in the past 5 years’, was one of the variables selected in this study as a strong predictor for not receiving rehabilitation in home care. On the other hand, this variable is not included in the decision algorithm on the CA. The fact that cancer was a top predictor for not receiving rehabilitation services may indicate that rehabilitation is seen as inappropriate for some persons with a terminal illness such as cancer. While additional research is needed in this area, especially for older patients with advanced disease [50], there is evidence that elderly patients with cancer – though they often do not receive rehabilitation services – are able to achieve both physical and mental functional improvement following rehabilitation [54–57].

As an indirect way to explore for potential interaction effects, we compared the rank order of the items using two different machine learning techniques. As described in the “Methods” section, variables that scored highly on the RF-VIM scale but not using the LASSO approach may be due to a certain interaction between them which can have a significant effect on the outcomes.

A limitation of this study is that, because the full RAI-HC is not administered to all clients who receive the CA, we cannot perform variable selection using data collected for the broader set of clients targeted by the CA at the point of intake into home care. In other words, variables such as the IADLs may have greater discriminant validity in a more heterogeneous population with greater diversity in functional status. We believe that our use of the frequency-matched dataset was the best available strategy to address this potential bias; however, some systematic differences between the two populations may still remain. Nonetheless, we feel these analyses provide useful insight into how actual clinical practices differed from the results of the process used to develop the RA. Research using a larger matched sample and follow-up with patients receiving both the CA and the RAI-HC could be used to further explore the issues discussed in this paper.

## Conclusion

While there is considerable evidence for the feasibility and effectiveness of home-based rehabilitation for older persons, many who could benefit do not receive needed rehabilitation services. Standardized assessment instruments, such as the RAI-HC used with home care clients in Ontario and other jurisdictions, have the potential to guide appropriate care planning and allocation of limited rehabilitation resources. In this study, we explored patient characteristics that predict rehabilitation services use in home care clients and compared them to what the interRAI consortium has defined as important characteristics to consider when addressing need in this group. We found that, while the two sets of characteristics agreed with each other to a certain extent, there are also some notable differences, especially with regard to cognitive status and cancer diagnosis. Our findings also suggest that data mining methods, such as the LASSO and the random forest, can play an important role in selecting important client characteristics for care planning.

## Abbreviations

CA: Contact assessment
HC: Home care
interRAI HC: interRAI home care
interRAI CA: interRAI contact assessment
RA: Rehabilitation algorithm
ISD: Instrument and systems development
VIM: Variable importance measure
RF: Random forest
ADL: Activities of daily living
IADL: Instrumental activities of daily living
